# Coming of Age for Autotaxin and Lysophosphatidate Signaling: Clinical Applications for Preventing, Detecting and Targeting Tumor-Promoting Inflammation

**DOI:** 10.3390/cancers10030073

**Published:** 2018-03-15

**Authors:** Matthew G.K. Benesch, Iain T.K. MacIntyre, Todd P.W. McMullen, David N. Brindley

**Affiliations:** 1 Discipline of Surgery, Faculty of Medicine, Memorial University of Newfoundland, St. John’s, NL AlB 3V6, Canada; mbenesch@mun.ca (M.G.K.B.); itkm80@mun.ca (I.T.K.M.); 2 Signal Transduction Research Group, Cancer Research Institute of Northern Alberta, Department of Biochemistry, Faculty of Medicine and Dentistry, University of Alberta, Edmonton, AB T6G 2S2, Canada; 3 Department of Surgery, Faculty of Medicine and Dentistry, University of Alberta, Edmonton, AB T6G 2G7, Canada; todd.mcmullen@ualberta.ca

**Keywords:** lysophosphatidic acid, lipid phosphate phosphatases, chemoresistance, radiotherapy, metastasis, fibrosis, chronic inflammation, hallmarks of cancer, adjuvant therapy

## Abstract

A quarter-century after the discovery of autotaxin in cell culture, the autotaxin-lysophosphatidate (LPA)-lipid phosphate phosphatase axis is now a promising clinical target for treating chronic inflammatory conditions, mitigating fibrosis progression, and improving the efficacy of existing cancer chemotherapies and radiotherapy. Nearly half of the literature on this axis has been published during the last five years. In cancer biology, LPA signaling is increasingly being recognized as a central mediator of the progression of chronic inflammation in the establishment of a tumor microenvironment which promotes cancer growth, immune evasion, metastasis, and treatment resistance. In this review, we will summarize recent advances made in understanding LPA signaling with respect to chronic inflammation and cancer. We will also provide perspectives on the applications of inhibitors of LPA signaling in preventing cancer initiation, as adjuncts extending the efficacy of current cancer treatments by blocking inflammation caused by either the cancer or the cancer therapy itself, and by disruption of the tumor microenvironment. Overall, LPA, a simple molecule that mediates a plethora of biological effects, can be targeted at its levels of production by autotaxin, LPA receptors or through LPA degradation by lipid phosphate phosphatases. Drugs for these applications will soon be entering clinical practice.

## 1. Introduction—Cancer as the Ultimate Disease of Chronic Inflammation

Rudolf Virchow, known as the father of modern pathology, observed under a microscope a high concentration of leukocytes in neoplastic tissues. He proposed in 1863 that the “lymphoreticular infiltrate” reflected the origins of cancer at sites of chronic inflammation [1]. His discovery however remained largely ignored for over a century. In that time, there was an ebb and flow of numerous ideas behind the origins of cancer, including viruses, environmental exposures, and genetic factors. These various ideas have at times pitted scientists against each other in debate as to the origins of cancer. However, given the vast heterogeneity of this disease, it is now recognized that they all are indeed correct for particular cancers. During the 1990s, after over a century of intense investigation, our knowledge about what cancer is as a disease began to coalesce, and it was codified and revised by Hanahan and Weinberg as the hallmarks of cancer [2,3]. Cancer is a disease of sustained proliferative signaling, evasion of growth suppressors, replicative immortality, angiogenesis, resistance to cell death, deregulation of cellular genetics, avoidance of the immune system, and invasion and metastasis [2,3]. These traits are enabled through two main characteristics common to virtually all cancers: genomic instability and mutation, and tumor-promoting inflammation [3].

**Figure 1:**
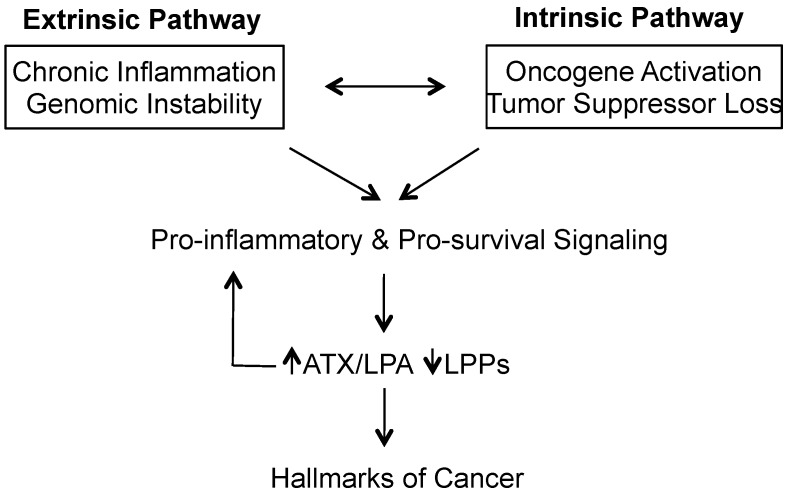
General overview of cancer initiation and the role of autotaxin/lysophosphatidate (ATX/LPA). A convergence of extrinsic and intrinsic pathways leads to sustained inflammatory and survival signaling that involves upregulation of ATX/LPA signaling through both increases in ATX and LPA concentrations with concurrent decreases in eco-lipid phosphate phosphatase (LPP) activity. The establishment of this vicious cycle leads to cancer initiation and progression as often described by the hallmarks of cancer (sustained proliferative signaling, evasion of growth suppressors, replicative immortality, angiogenesis, resistance to cell death, deregulation of cellular genetics, avoidance of the immune system, and invasion and metastasis) [3,6,7].

As Virchow noted, virtually all neoplastic lesions contain immune cells, and it has been long recognized by pathologists that these tumor-associated cells come from both the innate and adaptive arms of the immune system [4]. This immune cell composition is also present in inflamed non-neoplastic tumors, suggestive that the immune system actively attempts to destroy pre-malignant and early cancer cells, at least initially [4]. However, if there is no resolution of the injury, this milieu of perpetually smoldering inflammatory signaling (wounds that do not heal) is exploited by these neoplastic cells to complete the transformation into an established cancer [4,5]. This connection between chronic inflammation in many diseases such as hepatitis and inflammatory bowel diseases and their eventual transformation into cancers, has been coined the extrinsic pathway [6,7] (Figure 1). In this chronic inflammatory state, reactive oxygen species and other mutagenic molecules initiate genetic transformations leading to oncogene activation and loss of tumor suppressor functionality, termed the intrinsic pathway [6,7] (Figure 1). Together, these two pathways serve to upregulate a host of pro-inflammatory transcription factors, namely nuclear factor-κB (NF-κB), signal transducer and activator of transcription 3 (STAT3) and hypoxia-inducible factor 1α (HIF1α) in cancer cells [6]. These same signaling pathways also serve to promote cell survival and evasion from the immune system [6,8] (Figure 1). The overall net result is an increase in the production of cytokines and chemokines by neoplastic cells that spills over into adjacent stromal cells, resulting in more inflammatory mediators being produced, with further leukocyte activation and recruitment [6,9]. Hence, tumor-promoting inflammation both enables and is a product of genetic instability, and accentuates the other hallmarks of cancer [9]. Consequently, there is at least an 80% correlation between tumor leukocyte density and poor patient prognosis [10], with at least 20% of all cancer deaths being linked to underlying inflammatory processes [6]. 

Over the last decade, it has become well acknowledged that cancer can no longer be considered a disease of just cancer cells, but instead is a disease of unchecked cell growth and spread within a permissive host environment [11]. Now, in the age of targeted therapies, modern cancer therapy regimens need to disrupt the interactions between cancer cells and supportive stromal elements to mitigate both pro-inflammatory and pro-survival signaling and development of therapy resistance [12]. Appreciating and targeting chronic tumor-promoting inflammation through adjunct therapy may ultimately be the biggest breakthrough in cancer treatment in the next decade [13], and may be the most effective modality for cancer prevention, improving cancer therapy efficacy and maintaining remission. 

In this review, we propose that targeting chronic inflammation through inhibition of the autotaxin-lysophosphatidate-lipid phosphate phosphatase (ATX-LPA-LPP) axis provides one viable strategy for achieving this breakthrough. As potent anti-inflammatory agents, such inhibitors have the potential to be powerful adjuvant agents for improving the efficacy of cancer treatment and preventing therapy resistance irrespective of the genomic instability within cancer cells [14,15,16]. In the last five years, the literature on LPA signaling axis has nearly doubled. With the advent of multimodality clinical trials for targeting the ATX-LPA-LPP axis in chronic inflammatory diseases, we will summarize new findings to complement our previous reviews on this subject [15,16,17].

## 2. Overview of LPA Signaling and Its Importance in Reproduction and Embryonic Development

**Figure 2:**
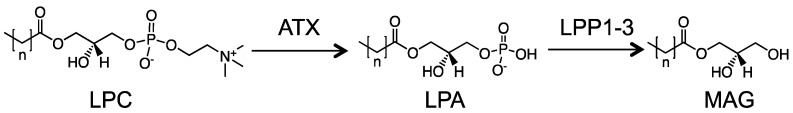
Overview of the LPA signaling axis. Extracellular LPA is produced from LPC by the lysophospholipase D activity of ATX. LPA then signals through at least six known G-protein coupled LPA receptors to mediate its host of physiological and pathological effects. LPA is rapidly turned over by the eco-activity of LPP1-3 into inorganic phosphate and monoacylglycerol (MAG), which apart from 2-arachidonoylglycerol, does not affect signaling.

As the simplest bioactive glycerolphospholipid, LPA contains a glycerol backbone with a phosphate head group, and an acyl chain (usually unsaturated) at the sn-1 (or sn-2) position (Figure 2). Extracellular LPA is generated primarily from lysophosphatidylcholine (LPC) in plasma by the lysophospholipase D activity of ATX, as demonstrated by ATX heterozygote mice having half-normal LPA levels [18,19]. ATX inhibition provides >95% reduction of LPA levels in plasma [20]. Conditional gene knockout of ATX in adipose tissues in mice results in up to a 38% decrease in circulating LPA levels, thus demonstrating the importance of adipose tissue in ATX secretion [21]. Some LPA, primarily saturated species, is also produced by hydrolysis of a fatty acid chain from the membrane-derived phosphatidate via phospholipase A1 and A2 activity in inflammatory cells, activated platelets and endothelial cells [22]. LPA mediates its plethora of effects by signaling through at least six G-protein coupled LPA receptors (LPA_1–6_) [17]. There are other orphan G-protein coupled receptors that may facilitate LPA signaling, including GPR35 [23], GPR87 [24,25], P2Y10 [26], and the receptor for advanced glycation end products (RAGE) [27]. LPA is turned over rapidly (t_1/2_ of ~1 min) in plasma into monoacylglycerol and inorganic phosphate by the ecto-activity of three lipid phosphate phosphatases (LPP1-3) [17,28] (Figure 2). 

This ecto-activity helps regulate the total LPA pool, and in particular reduces LPA concentrations in the immediate cellular microenvironment [29]. This may in part explain why cancer cells, which become dependent on sustained LPA signaling, normally have decreased LPP expression (see Section 6). We refer readers to several other recent reviews for a more detailed description of LPA receptor and LPP biology [29,30,31,32,33].

ATX, an ectonucleotide pyrophosphatase/phosphodiesterase (ENPP) enzyme, is encoded by the ENPP2 gene. This is one in a family of seven mammalian enzymes that belong to the superfamily of ecto-nucleotideases that hydrolyze pyrophosphate and phosphodiester bonds of a wide range of nucleotides and their derivatives [34,35]. ATX is unique in having a specific lysophospholipase D activity owing to its additional hydrophobic binding pocket that interacts with the acyl chain of lysophospholipids [36]. As a secreted glycoprotein of about 125 kDa, full-length ATX is synthesized as a pre-proenzyme and is secreted by the classical secretory pathway under the influence of Akt-signaling [37,38]. ATX was first discovered in A2058 melanoma cell culture in 1992 and it was later shown to hydrolyze LPC preferentially [39,40]. Five isoforms of ATX have since been discovered in human beings [41,42]. Despite this, under physiological conditions, all isoforms have largely similar kinetic effects and thus their biological significance is unknown [42]. Nevertheless, all critical residues and structural elements are highly conserved between mice and human beings, underscoring its physiological importance [43]. 

In relation to reproduction, endometrial stromal cells in culture are highly sensitive to LPA signaling as demonstrated by growth inhibition with either ATX or LPA receptor inhibitors. The source of this LPA is from autocrine-produced ATX [44]. Multiple LPA receptors are likely involved since equal proliferation occurs in endometrial cells with either LPA_1_ or LPA_3_ knockouts compared to wild type cells [44]. Strong correlations have also been found in animal models between ovarian follicular growth and LPA signaling [45,46]. ATX is also a tissue-remodeling factor in regressing corpora lutea [47]. Another study showed that placental transcription of ATX increases progressively during normal pregnancy, and a disturbance in placental ATX production is linked to early pre-eclampsia [48]. Serum ATX levels show a significant correlation with maternal blood pressures and uric acid levels, both of which are parameters for the severity of pre-eclampsia [49]. Targeting ATX/LPA signaling could limit pregnancy complications associated with pre-eclampsia, and this represents an emerging field where LPA-modulating therapies could hold significant therapeutic promise.

It has been known for over a decade that complete knockout of ATX in mice is embryonically lethal at day 9.5, secondary to profound vascular and neural crest defects [18,19]. Later work showed that this lethality was due to the absence of ATX catalytic activity, since a single point mutation of the catalytically active amino acid threonine-210 to alanine produces the same lethal phenotype [50]. Ex vivo, the neural defects were partially ameliorated by addition of LPA to culture [51]. In zebrafish, ATX knockout studies have shown that the ATX-LPA-histone deacetylase (HDAC)1/2 axis regulates oligodendrocyte differentiation in the hindbrain [52,53], and that ATX/LPA_3_ signaling regulates left-right asymmetry [54]. Therefore, ATX-mediated signaling in neurological development is an evolutionally conserved mechanism from at least zebrafish to rodents [53].

More recently, ATX overexpression in mouse embryos was demonstrated to be lethal at around day 9.5–11.5 with growth retardation, open and kinked neural tubes, abnormal allantois, and vascular defects [55]. In this same study, induced overexpression of ATX in the neonatal period caused vascular instability, a delay in retinal vascularization, and a decrease in vessel branching [55]. Interestingly, LPP3 knockout in mice is also embryonically lethal with very similar vascular defects to those embryos overexpressing ATX [56,57]. Like the knockout studies, overexpression of ATX in the zebrafish embryo leads to distortion of axial midline and left-right asymmetry in the embryo through the Rho/ROCK pathway via LPA_1–3_ signaling. [58]. Overexpression via injection of ATX messenger RNA (mRNA) in zebrafish embryo also induces cardia bifida [59]. Therefore, it appears that LPA concentrations must be tightly regulated to ensure proper development. This is especially apparent from mouse models, as both ATX knockout and overexpression are embryonically lethal [18,19,55]. With respect however to LPA receptor signaling, there is significant redundancy since no individual, double or triple LPA receptor knockout mouse model is lethal [60]. 

## 3. LPA Signaling in Wound Healing and Immunity 

In the post-natal organism, one of the most important roles of the ATX-LPA-LPP axis is mediating wound healing and tissue remodeling. LPA is a potent activator of platelet aggregation and it promotes the growth and migration of immune cells, fibroblasts, endothelial cells and keratinocytes into sites of tissue damage. The mechanisms for these effects have been reviewed elsewhere [17,61,62,63]. 

There have been multiple recent advances in LPA biology, most of which relate to immune system regulation and tissue repair. In the innate immune system, LPA promotes lymphocyte extravasation, which maintains immune homeostasis by stimulating the conversion of CD11b+ murine monocytes into F4/80 macrophages. A similar effect also occurs in human beings through a common transcription factor, peroxisome proliferator-activated receptor (PPAR)γ [62]. ATX is induced in immune cells in response to toll-like receptor activation by lipopolysaccharide, via a type I interferon autocrine-paracrine loop involving the JAK-STAT and PI3K-Akt pathways [64]. ATX is highly expressed in lymph node stromal cells promoting interstitial T cell migration in lymph nodes primarily through LPA_2_ signaling [65,66,67]. LPA promotes and converts primed pluripotent stem cells into naïve stem cells through LPA_1_-STAT3 signaling pathways, which has implications in regenerative medicine [68]. 

Our understanding of the role of LPA in neuronal inflammation and regeneration is also evolving and demonstrates new complexities in LPA receptor signaling not previously well appreciated. Serum LPA concentrations and ATX levels in the central nervous system are decreased in patients with multiple sclerosis (MS) compared to healthy controls [69,70]. LPA levels are restored during symptom-free and recovery intervals in murine models of experimental autoimmune encephalomyelitis (EAE), which mimic MS [69]. These influences appear to be mediated through LPA_2_ since there is more intense disease in LPA_2_-deficient mice, and recovery in wild-type mice is improved with LPA_2_ agonist therapy [69]. This recovery appears to be mediated by increasing T-cell homing and promoting remyelination [69]. This is in line with other work demonstrating that reductions in ATX-LPA-HDAC1/2 signaling may contribute to MS pathology and that LPA is important for oligodendrocyte maturation [53,71]. However, significant reduction of total LPA concentrations with the ATX inhibitor Compound-1 resulted in deceased disease severity in a very similar murine model of EAE [72]. These two contrasting studies highlight the emerging complexity of LPA receptor-mediated signaling. While LPA_2_ signaling may limit disease intensity, LPA signaling through other receptors may have the opposite effect, meaning that disease burden could be dependent on subtle changes in receptor abundance. 

In both zebrafish and mice, ATX-LPA_1_ signaling contributes to proliferation of chondrocytes by promoting S-phase entry and regulating fibronectin assembly, leading to proper cartilage formation [73]. Finally, C18:1-LPA signaling may be a master transcriptional regulator of primary human gingival fibroblasts that mediate gingival repair, but under chronic inflammatory conditions it promotes periodontal disease [74,75,76].

The induction of ATX by acute inflammation in tissue repair has been well appreciated for many years, but the regulation of this process is only now starting to emerge. We showed that LPA normally downregulates ATX production at the mRNA level through LPA_1_-PI3K signaling [77]. This suppression is overcome via parallel induction of ATX synthesis by signaling through inflammatory cytokines such as TNFα and IL-1β [77]. ATX is eliminated rapidly from the circulation through a scavenger receptor-mediated process in liver sinusoidal endothelial cells [78]. Therefore, once this inciting injury is resolved, basal expression of ATX, and in turn LPA concentrations, are restored [77].

## 4. Maladaptive LPA Signaling in Chronic Inflammatory Diseases

This regulation of ATX turnover by inflammatory cytokines also explains why elevated ATX and LPA levels persist in chronic inflammation. In addition, it is also well established that LPP1 and LPP3 are downregulated in cancers, which aggravates the increase in LPA levels caused by the elevated ATX [29]. An emerging hallmark of chronic inflammation is sustained LPA production and signaling, leading to propagation of the disease phenotype. These pathologies are now being elucidated in vivo in conditional ATX tissue knockouts, especially in models of arthritis, pulmonary fibrosis and liver diseases [79,80,81]. The chronic inflammatory states in many of these conditions often progress into cancer, as will be discussed in the next section. Below, we summarize major recent advances in LPA signaling in chronic inflammation.

### 4.1. ATX and LPA in Inflamed Adipose Tissue and the Metabolic Syndrome

There is considerable evidence that LPA contributes to the chronic low-level inflammatory state associated with a myriad of complications tied to obesity, insulin resistance, diabetes, dyslipidemia, atherosclerosis, and hypertension in the Metabolic Syndrome [82]. About 40% of the ATX in mice is produced by adipose tissue, and this increases further with increased dietary fat consumption [83]. This increased ATX secretion is compounded by the expansion of total adipose tissue mass in obesity. Inflammation of adipose tissue also increases ATX expression [77]. Patients with type 2 diabetes have higher ATX levels in serum and visceral fat than non-diabetic patients, and ATX correlates positively with body fat percentage and increased leptin/adiponectin ratios. This correlates negatively with glucose clearance rates [21,84,85]. Leptin and adiponectin have opposing effects on low-grade adipose tissue inflammation and insulin resistance, with leptin upregulating proinflammatory cytokines, and adiponectin having the opposite effect [86]. Thus, the leptin/adiponectin ratio is being increasingly positively correlated to the co-morbidities of the Metabolic Syndrome [87,88]. Similarly, serum ATX is an independent predictor of insulin resistance in older obese adults [89]. In mice lacking ATX expression in white adipose tissue, adiponectin and GLUT-1 levels were increased, leading to increased glucose tolerance [21]. Transgenic mice overexpressing ATX exhibit reduced expression of thermogenic brown adipose tissue in exchange for peripheral white adipose tissue, and have increased weight gain when fed a high fat diet [90]. 

LPA is able to attenuate insulin signaling in rat hepatocytes via LPA_3_ signaling through decreased hepatocyte glucose uptake and glycogen synthesis [91]. In the same study, the authors reported LPA 16:0 concentrations to be about 25 percent higher in obese patients with a body mass index (BMI) >30 compared to normal weight individuals (BMI 18.5–25) [91]. ATX expression in adipocytes has recently been shown to be upregulated through the glycoprotein 130 (gp130)-mediated Janus kinase (JAK)-STAT3 pathway via IL-6 family cytokines [92]. Treating high-fat diet-fed obese mice with the oral gp130 inhibitor SC144 suppressed adipose tissue ATX expression, resulting in decreased plasma ATX, LPA and free fatty acid levels, and increased glucose tolerance [92].

While there is strong in vivo and clinical evidence supporting the effect of ATX/LPA in adiposity, there is still some controversy in the strength of the association. This is because a negative relation between BMI and ATX expression levels has been reported in both adipose tissue and serum of non-diabetic human subjects [93]. However, in the same report, serum ATX levels tended to be higher in diabetic subjects [93], and most evidence supports increased ATX/LPA levels in diabetics [84,94]. These discrepancies might in part be rationalized by the product inhibition of LPA on ATX expression under physiological conditions [77]. While increased adipose tissue may lead to increased ATX production, the subsequent increase in LPA concentrations acts as a rheostat to restore balance, leading to eventual decreased ATX levels. However in a more pro-inflammatory condition, such as diabetes, the additional cytokine stimulation of ATX production is enough to overcome this inhibition, a phenomenon best illustrated in cancer models [77,95]. Further delineation of ATX/LPA signaling pathways in obesity is currently a very active area of research. 

Several authors have linked LPA signaling to cardiac diseases. MMP-9 is particularly associated with rupture of atherosclerotic plaques in coronary arteries, and LPA upregulates MMP-9 expression through LPA_2_ signaling and nuclear factor-κB (NF-κB) pathways in macrophages [96]. Fasting plasma ATX concentration is a novel independent predictor of calcific aortic valve stenosis in patients with coronary artery disease [97,98]. Mechanistically, ATX is transported to the aortic value by apolipoprotein (a) and is secreted by valve interstitial cells, leading to inflammation and calcification [99]. In contrast, LPP3 negatively regulates aortic endothelial cell inflammation and could mitigate the development and complications of atherosclerosis [100]. LPP3 silencing in human primary aortic endothelial cells enhances cytokine secretion and leukocyte adhesion whileimpairing angiogenesis, whereas LPP3 overexpression reverses these effects [101,102]. Similar effects occur via pharmacological inhibition of both LPA and sphingosine 1-phosphate signaling, both substances that are degraded by LPP3 [101]. 

### 4.2. ATX and Neurological Disorders

LPA also acts synergistically with adipokines (leptin, resistin, TNFα, IL-1β and IL-6) released by white adipose tissue in promoting inflammation and producing reactive oxygen species that disrupt blood brain barrier permeability, leading to hippocampal atrophy and dementia development [103]. Significantly, increased ATX levels are associated with a 3.5- to 5-fold risk of mild cognitive impairment and Alzheimer’s disease [104]. Other conditions well reviewed elsewhere include neuropathic pain secondary to inflammation-mediated demyelination of axons [105,106,107]. 

### 4.3. ATX and Arthritis

ATX concentrations in plasma and synovial fluid correlate with the severity of knee osteoarthritis [108]. Mice fed high fat diets exhibit both accelerated surgically-induced and age-related osteoarthritis [109]. In these mice, leptin contributes to metabolic and catabolic changes in articular cartilage, resulting in local increased ATX expression. This in turn promotes cartilage degeneration by increased MMP-13 production, which is the major catabolic enzyme responsible for cleaving type II collagen in cartilage [109]. ATX and MMP-13 are significantly increased in leptin-treated chondrocyte cultures compared to vehicle treated cultures and inhibition of ATX activity decreased leptin-induced MMP-13 expression [109]. Increased ATX levels and LPA signaling are also involved in rheumatoid arthritis, in part by promoting synovial hyperplasia, which lead to progressive destruction of cartilage and bone [17,110].

### 4.4. ATX and LPA in Pulmonary Fibrosis and Asthma

LPA-mediated lung fibrosis is a very established field of research for which at least two LPA receptor antagonists and an ATX inhibitor have entered clinical trials [16] (Section 7). The foundational animal studies behind these clinical trials demonstrated in a bleomycin-induced mouse model of lung fibrosis that fibroblast migration depends on LPA_1_ signaling. Mice that lacked LPA_1_ were more resistant to fibrosis via bleomycin challenge [111,112]. In response to bleomycin, ATX in bronchoalveolar lavage fluid increased 17-fold and this ATX appeared to be of plasma origin that enters the alveolar space via vascular leak [113]. In mouse models of lung allograft fibrosis, mesenchymal cells from fibrotic lung allografts have constitutive β-catenin expression [114]. β-catenin expression depends strongly on autocrine ATX signaling, and both ATX and β-catenin are regulated by the transcription factor nuclear factor of activated T-cells (NFAT1) [114]. Consequently, inhibition of LPA_1_ or ATX, either genetically or pharmacologically, limits the development of fibrosis through decreased active β-catenin and dephosphorylated NFAT1 expression levels [114]. In another model of hyperoxic lung injury, capillary leakage and respiratory distress were limited by Brp-LPA, a commonly used pan-LPA receptor/ATX inhibitor [115]. Other studies, which were reviewed previously, have associated asthma with upregulation of ATX expression [17,111]. This has now been shown to be mediated primarily through LPA_2_ signaling [111]. Polyunsaturated LPAs, primarily C22:5-LPA and C22:6-LPA, are being investigated as potential biomarkers for the severity of allergy-mediated airway inflammation and disease in asthma [116]. 

### 4.5. ATX and LPA in Autoimmune and Retinal Diseases

Other chronic inflammatory and autoimmune diseases that are now recognized to be mediated by LPA signaling, include Sjogren’s syndrome, a condition causing dry eyes and mouth. In mice, Sjogren’s syndrome is exacerbated by LPA_1/3_-mediated production of IL-17 in a dose-dependent manner. Saliva volume is restored by treatment with the LPA_1/3_ antagonist, Ki16425 [117]. In retinal disease, macular edema is medically treated with steroids and anti-vascular endothelial growth factor (VEGF) agents [118]. LPA and ATX concentrations are positively correlated with the proinflammatory mediators, IL-6, IL-8, MCP-1 and VEGF in patients with macular edema secondary to retinal vein occlusion [119], this finding opens the possibility for ATX/LPA targeted therapy.

### 4.6. ATX and LPA in Hepatic Diseases

Pathogenic LPA signaling is increasingly implicated in a host of hepatic disease conditions. The severity of one of these conditions, biliary atresia, a progressive fibro-inflammatory liver disease, correlates independently with both liver stiffness and serum ATX levels [120]. In a later follow-up report, and the first to study ATX promoter methylation, DNA was extracted from both circulating leukocytes and liver tissues of biliary atresia patients and age-matched controls [121]. Patients with biliary atresia have decreased ATX promoter methylation that was even lower in patients with advanced disease [121]. Consequently, relative ATX mRNA and serum protein levels are both inversely correlated with overall ATX methylation level and positivity associated with jaundice status, hepatic dysfunction and liver stiffness [121]. Mechanistically, fibrosis by LPA signaling is a two-step process whereby ATX expression is largely confined to hepatocytes in response to injury, and LPA_1_ is highly expressed in the collagen-secreting hepatic stellate cells [122]. Patients with chronic liver disease of various origins (viral, alcoholic, or fatty liver) have both increased ATX and LPA_6_ expression [81]. This same pattern has been previously correlated to tumorigenicity in hepatocellular carcinoma [123].

Serum ATX may also be a potential biomarker for non-alcoholic fatty liver disease (NAFLD) in obese non-diabetic women. This correlates significantly with other markers of NAFLD including alkaline phosphatase and fasting glucose, insulin and triglyceride levels [124]. 

Many observational studies and reviews have linked ATX to cholestatic pruritus and liver injury [125,126]. In the first prospective study of its kind, patients with primary biliary cholangitis and primary sclerosing cholangitis were followed for 60 months [127]. Serum ATX was significantly higher in both groups compared to controls and in patients with either cirrhosis or longer disease duration [127]. ATX activity correlated strongly with Model for End-Stage Liver Disease (MELD) scores, Mayo Risk scores, and worse disease-specific health-related quality of life (HRQoL) aspects [127]. High ATX levels are a negative predictor of survival, with such patients at a 2.6- to 4-fold increased risk of death or of requiring liver transplantation [127].

Chronic viral hepatitis infection that has progressed to cirrhosis accounts for 70% of all hepatocellular carcinomas, which represents 90% of all liver cancers [128]. Serum ATX is a superior marker for predicting significant fibrosis in both chronic hepatitis B and C patients. This can be analyzed without consideration of food intake prior to phlebotomy [129]. More recent studies of both hepatitis B and C showed that serum ATX was the most reliable marker for all fibrosis stages compared with other serum markers including hyaluronic acid, type IV collagen 7S, aspartate aminotransferase-to-platelet ratio and FIB-4 index [130,131]. Mechanistically, hepatitis C infection stabilizes hypoxia inducible factors, which in turn increased hepatocellular ATX expression. This cycle is reinforced since LPA signaling through PI3K stabilizes hypoxic inducible factor (HIF)-1α [132]. Elevated serum ATX levels in patients with hepatitis C or co-infection with HIV partially normalize within 6 months of starting interferon-free hepatitis C therapy [133]. Therefore, ATX has significant potential to be both a diagnostic and therapeutic target in mitigating the progression of viral hepatitis into cancer [128,134].

### 4.7. Importance of the Site of ATX Production in Inflammatory Signaling

One of the outstanding problems in understanding the physiological and pathological roles of ATX secretion is whether signaling by LPA is regulated locally rather than by globally produced ATX. It was established from structural studies of ATX that it binds locally to integrins on cells surfaces [63,135] and that this delivers LPA to signal through nearby receptors [36]. The reports that ATX expression in specific cells, such as adipocytes, fibroblasts, hepatocytes, and lung parenchyma, leads to pathology in different organs [79,80,81,136,137] adds weight to the concept that it is locally produced ATX that has a major signaling role. Locally produced ATX interacting with integrins on nearby cells is likely the primary means by which inflammation in wound healing and tissue repair is localized to the site of injury. However, ATX is just as catalytically active when not bound to integrins [138], which may also explain why plasma LPA levels are increased in numerous disease processes [77,124,133]. Overall, the function however of plasma ATX is still uncertain: it could represent transport among organs and/or the ATX that is en route for hepatic degradation.

## 5. Recent Developments in LPA Signaling in Cancer Biology

Since ATX was first discovered in melanoma cell culture, ATX and LPA signaling have been associated with cancer initiation and progression, survival against cancer therapy, and metastasis for virtually every major cancer type [17,139]. The common theme is that chronic inflammation upregulates ATX production by either the cancer cells themselves or in adjacent tumor stroma. This is often accompanied with a concomitant overexpression of LPA receptors, particularly LPA_1–3_, and a downregulation of the ecto-activities of the LPP1/3 [17]. The overall net effect of sustained LPA signaling is to increase angiogenesis and cancer cell growth, migration, and survival. 

### 5.1. Mechanisms of ATX Overexpression in Cancers

For those cancers that secrete ATX, several mechanisms of overexpression are now appreciated. Most genomic alterations in the ATX gene are copy number amplifications [22,35]. In other cases, ATX is over-expressed in response to DNA double-strand breaks secondary to oxidative stresses in cancer cells. This depends on activation of the NLRP3 inflammasome and ataxia telangiectasia mutated (ATM) phosphorylation. The ATM-ATX-dependent loop further propagates inflammation and additional double strand breaks, leading to further ATX production [140]. The first study to look at post-transcriptional regulation of ATX demonstrated that the RNA-binding protein ELAV-like protein 1 or human antigen R (HuR) enhances ATX mRNA stability in melanoma cells and thus increases ATX production [141]. LPA signaling in turn increases HuR protein expression [141]. HuR stabilizes the mRNA for many pro-inflammatory proteins and it is itself a prognostic factor for poor outcome in ovarian and breast cancers [142,143]. We showed previously that ATX is significantly upregulated in thyroid cancer in response to sustained autocrine-induced inflammation, and this can distinguish cancer from benign disease [144]. Since then, HuR has shown to be overexpressed in thyroid cancers but not benign nodules [145]. Taken together, these findings suggest that copy number amplifications and inflammatory-induced mRNA stability resulting in increased ATX translation are likely major mechanisms behind autocrine ATX production in cancers.

A seminal study showed in a mouse mammary tumor virus (MMTV) model that overexpression of ATX or any of LPA_1-3_ increased mammary tumorigenesis, invasion and metastasis [146]. However, breast cancer cells secrete little ATX compared to the basal rate from breast adipose tissue [137]. We reconciled this discrepancy and uncovered a paracrine-model of ATX production in breast tumors. This involved an immunocompetent orthotopic murine breast cancer model. We demonstrated that secretion of inflammatory mediators from the breast tumor induce ATX expression in adjacent mammary tissue. This subsequently increases LPA within the tumor microenvironment [137,147] and it establishes a vicious loop of inflammatory-driven ATX production since LPA increases the production of more inflammatory cytokines and COX-2. This cycle can be broken with a potent ATX inhibitor (Section 7). Likewise, tumor-induced inflammation in mammary adipose tissue increases macrophage recruitment, which leads to further inflammation and ATX production [137]. These bidirectional interactions between breast cancer cells and ATX production in mammary adipose tissue have since been confirmed [148]. Increased ATX production in adipose tissues could provide a possible link between obesity and its contribution to an estimated 20–40% of breast cancers [149,150].

### 5.2. LPA Signaling in Cancer Progression

Hepatocellular carcinoma (HCC) is well established to be associated with LPA signaling. HCC has a 5-year survival rate of less than 15% primarily due to late detection and poor screening [122]. HCC is usually a consequence of chronic inflammation from viral hepatitis and patients are at an eight-fold increased risk of death if ATX levels are elevated [151]. Higher LPA_2_ mRNA levels in HCC specimens are correlated with poorer differentiation, and higher LPA_6_ mRNA levels are correlated with microvascular invasion. These observations suggest an overall higher malignant potential with increased LPA_2/6_ expression [152]. This study, like others, correlated ATX with disease progression, but more interestingly showed that the mRNA level of phospholipase A1α, another LPA producing enzyme, has no pathological association [152]. Further, hepatocyte-specific ATX-deficient mice were protected from fibrosis and HCC development compared to controls [81]. Even with the decreasing incidence of Hepatitis B and C infections with more efficacious therapies, NASH will become the primary cause of HCC in the western world by 2030 because of increasing obesity rates [153]. Given the well-established roles of LPA signaling in the pathogenesis of metabolic-related syndromes, ATX/LPA signaling inhibitors should also have therapeutic benefit in HCCs from these origins.

Increased serum ATX activity was most significant for exocrine pancreatic cancer in an evaluation of serum ATX levels in a host of gastrointestinal cancers including cancers of the esophagus, stomach, colon, biliary tract and pancreas [22,154]. Exocrine pancreatic cancer is the fifth major cause of cancer death in the developed world, with a 5-year survival rate of less than 5% [155]. Therefore, serum ATX in combination with other biomarkers could one day offer an early diagnostic tool. However, serum ATX activity is not increased in patients with chronic pancreatitis or pancreatic cysts and neither condition is predictive of pancreatic cancer risk [154]. Lastly, in another study comparing the ascites of gastric cancer patients to those of cirrhosis, higher ATX and LPA levels were found in cirrhosis patients as expected [156]. However, higher lysophosphatidylserine and lysophosphatidylglycerol levels were found in gastric cancer ascites, which may act as substrates for LPA generation [156]. This suggests that glycerol-lysophospholipids other than LPA might be involved in pathogenesis of cancer directly or through being converted into LPA [156]. 

Advances in the LPA field have also been made in gynecological cancers. Ovarian cancers have a high mortality because they metastasize easily and through the development of resistance to chemotherapy [157]. These characteristics are mediated by cancer stem cells, which in ovarian cancer produce abundant ATX and they express high LPA_1_ [158]. Knockdown of either ATX or LPA_1_ leads to loss of stem cell markers and decreased tumorgenicity in xenografts [158]. Thus, the ATX-LPA-LPA_1_-Akt1 axis maintains cancer stem cell characteristics through an autocrine loop [158]. LPA_1_ and LPA_2_ are also involved in LPA-induced proliferation and angiogenesis in endometrial cancer tissue with positive correlations between LPA receptor and ATX protein levels with cancer stage [159].

In connection to other cancers, HT1080 fibroscarcoma cells that have been treated chronically with cisplatin have markedly elevated ATX and LPA_2_ expression and are much more mobile than controls [160]. Knockdown of LPA_2_ in these long-term treated cells reduced colony formation and autocrine production of ATX [160]. Multiple myeloma cells stimulate mesenchymal stromal cells to produce ATX [161]. In renal cell carcinomas, LPA activates Afr6-regulated mesenchymal pathways via LPA_2_ that promote cancer cell plasticity, metastasis and drug resistance [162]. Finally, several new studies have expanded our knowledge of LPA biology in melanoma, the first cancer linked to ATX. The transcription factor NFAT1 has roles in both innate and adaptive immune responses, and increases metastatic potential by directly upregulating IL-8 and MMP-3. This is secondary to autocrine ATX production and LPA signaling [163,164]. In work with the highly metastatic B16F10 murine melanoma, mice lacking either LPA_1_ or LPA_5_ had significantly less metastasis, indicating the importance of host LPA signaling for establishing a permissible environment for cancer cell seeding [165,166]. 

## 6. The Roles of LPPs in Cancers

Evidence is now mounting from experiments in vivo that increasing the low LPP1/3 expression in cancer cells limits tumor progression. Increasing LPP1 expression in syngeneic and xenograft breast and thyroid cancer models decreased tumor growth and metastasis by up to 80% through both increasing extracellular LPA degradation and decreasing the stimulation of Ca^2+^-transients by LPA [167]. Similarly, increasing the low LPP3 activity in SKOV3 ovarian cancer cells decreased tumor growth in nude mice [168]. Low expression levels of the LPP1 gene (*PLPP1*) has been identified as one of twelve markers predictive of poor breast cancer survival [169].

The effects of LPP2 on tumor growth are very different from LPP1 and LPP3. LPP2 expression is increased in breast, lung and ovarian cancers where the expression of LPP1 and LPP3 are decreased [29]. Overexpressing LPP2 in fibroblasts leads to premature S-phase entry, and this effect is not seen with LPP1/3 [170]. Knockdown of LPP2 delays entry into S-phase [170] and impairs anchorage-dependent growth [171]. These observations provide preliminary evidence that increasing the low expressions of LPP1 and LPP3 and decreasing the high expression of LPP2 in cancer cells could provide novel targets for cancer therapy. 

Our group discovered that one way of increasing LPP activity is through the use of tetracyclines, which increases the expression of the LPPs on the plasma membrane by enhancing LPP protein stability. This results in increased LPA clearance from circulation [28]. We then built on this finding to show that this doxycycline effect of increasing total ecto-activity of the LPPs in vivo also decreases circulating LPA concentrations, NF-κB translocation to the nucleus and IκB phosphorylation in breast cancer cells. This resulted in delayed breast tumor growth, deceased tumor macrophage infiltration and angiogenesis, and an overall reduction in the inflammatory milieu of the tumor environment [172]. Hence, tetracyclines and other potential activators of LPPs could become useful adjuvant therapies for decreasing signaling by LPA in cancers and other inflammatory diseases. 

## 7. Pharmacological Targeting of LPA-Mediated Inflammation and Cancer Progression

As extracellular targets, ATX and LPA receptor inhibitors are ideal pharmacological targets for a plethora of conditions [173,174,175]. Because of redundancies through LPA receptor signaling, much of the research has focused on the development of ATX inhibitors, thereby impeding LPA signaling through all receptors [17]. Numerous ATX inhibitors that are potent in vivo have been developed over the last five years, and it was important to assess the safety of these inhibitors, given the role of LPA signaling in mediating tissue repair and remodeling. Fortunately, the bulk of ATX in murine models is dispensable in the adult organism [176]. Conditional genetic ablation of ATX in the adult mouse shows no deleterious changes in liver, pancreatic or kidney function, and in blood cell populations [176]. High doses of the competitive ATX inhibitor, PF8380, produced no weight loss or histopathological changes in 13 major organs [176]. In the context of sustained ATX inhibition, the production of small amounts of LPA by other enzymes such as phospholipase A1 is likely to provide sufficient LPA for physiological functions, but not enough to drive pathology [15]. Recent progress made in pharmacological targeting of LPA signaling has now led to the first human clinical trials, where the inhibitors appear to be well tolerated (Section 7.4).

### 7.1. Limiting Fibrosis through Potent ATX Inhibition

Recent studies in vivo have shown that ATX inhibition by PAT-048 limited bleomycin-induced dermal fibrosis in mice, via inhibition of an ATX-LPA-IL-6 amplification loop. Similar results were obtained in cultures of dermal fibroblasts from patients with scleroderma fibrosis [177]. This same group showed prevention of fibrosis in the same models with LPA_1_ deletion or antagonism [178]. Consistently, toclizumab, a monoclonal antibody against the IL-6 receptor, showed success against systemic sclerosis in Phase II clinical trials [179]. In other fibrotic models, the progression of NAFLD to non-alcoholic steatohepatitis and liver fibrosis has been blocked in animal models with PAT-505, another novel ATX inhibitor [180]. Similar results have been obtained with the ATX inhibitors AM063 and AM095, which mitigated fibrosis progression and reduced development of HCC by disrupting validated HCC risk gene signatures in cirrhosis-driven HCC rat models [181]. With regard to lung pathologies, while ATX concentrations increased in bronchoalveolar lavage fluid following lipopolysaccharide exposure, neither genetic nor pharmacologic targeting or ATX or LPA receptors reduced the severity of acute lung injuries [182]. However, there is ample evidence including work in clinical trials supporting the efficacy of LPA inhibition in chronic lung conditions. This suggests that pharmacological targeting of LPA signaling is most effective in the context of persistent inflammation [182]. 

Beyond fibrosis, another competitive ATX inhibitor, Compound 1, exhibits a dose-dependent decrease in joint pain in multiple rodent models [183]. This same group also demonstrated anti-inflammatory and analgesic properties with Compound 1 in models of inflammatory bowel disease and multiple sclerosis [72]. In addition to chemical competitive inhibitors, anti-ATX DNA aptamers have also been designed, and the aptamer RB014 exhibits efficacy in a mouse bleomycin-induced model of pulmonary fibrosis [184]. Bile acids also act as partial non-competitive inhibitors of ATX suggesting that steroid-based drugs may have ATX antagonism properties [185]. 

### 7.2. Improving Cancer Chemotherapy Effectiveness by Blocking LPA-Mediated Inflammatory Signaling

Given the considerable overlap between fibrotic and carcinoma pathways [136], LPA signaling inhibitors are expected to serve as useful adjuncts in cancer therapy, largely by mitigating the loss of efficacy for chemotherapy and radiotherapy via blockage of the pro-survival benefits of tumor-promoting inflammation. These include limiting the physical barriers to drug deposition in tumors by desmoplastic reactions, which encase tumors in dense extracellular matrices, and vessel hyperpermeability, which compromises tumor accumulation of chemotherapeutics [11]. We have demonstrated in both thyroid xenograft and orthotopic mouse breast cancer models that the potent ATX inhibitor, ONO-8430506, reduces tumor growth and metastasis by up to 70%. This involves reducing the expression of up to 20 inflammatory cytokines and chemokines that drive tumorigenesis themselves and by further increasing ATX secretion and LPA concentrations [137,144,147]. ATX inhibition in turn is synergistic with doxorubicin treatment, in part by decreasing the LPA-induced expression of the transcription factor nuclear factor (erythroid-derived 2)-like-2 (Nrf-2), which activates the anti-oxidant response element. This promotes the synthesis of antioxidant proteins and multidrug-resistance transporters [186]. LPA thereby protects cancer cells from oxidative damage from most common therapies including tamoxifen, paclitaxel, cisplatin, and possibly ionizing radiation through increasing the effects of transcription factors like Nrf-2 [187,188].

### 7.3. Sensitizing Tumors to Radiation Therapy by Blockage of LPA Signaling 

LPA-mediated protection of cancer cells against radiation-induced cell death is partly mediated through LPA_2_ by stimulation of pro-survival kinase pathways that involve depleting cells of the proapototic signaling protein, Siva-1 [188]. ATM-mediated NF-κB activation occurs in response to radiation-induced DNA double-strand breaks, and plasma ATX and LPA levels increase. The resulting increase in LPA_2_ signaling accelerates the resolution of γH2AX histones [188]. Consequently, as predicted, LPA_2_-knockout mice are deficient in DNA damage repair mechanisms and have significant higher residual γH2AX histones after radiation exposure [188]. LPA also promotes the synthesis of numerous genes that promote DNA repair and this involves an increase in Nrf-2 expression [189,190]. Consequently, the Nrf-2 blockade has been proposed as an additional target for increasing the efficacy of radiation therapy [191]. Part of the increase in Nrf-2 expression could be mediated by the observed radiation-induced increased in the expression of ATX and LPA_1_ [192]. These observations that LPA protects against radiation-induced damage are compatible with studies showing that inhibition of ATX with BrP-LPA (also a pan-LPA receptor antagonist) or PF-8380 increased the sensitivity of heterotopic glioblastomas to radiation in mice [193,194]. 

**Figure 3:**
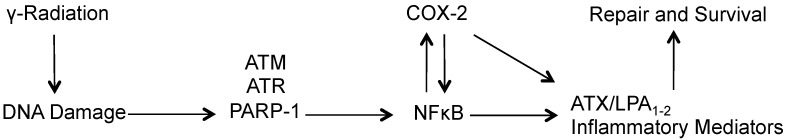
Overview of γ-radiation-induced inflammation in adipose tissue. γ-radiation, upon inducing double strand DNA breaks, activates the proteins ATM, ATR, and PARP-1. These in turn activate NFκB, facilitating the expression of COX-2, ATX, LPA_1–2_ and numerous inflammatory mediators, which in concert lead to repair and survival of tissues.

Treatment of breast cancer after the surgical removal of the tumor following lumpectomy commonly involves exposure of the whole breast to about 16-25 fractions of 2 to 2.65 Gy of radiation to eliminate remaining cancer cells [195]. Exposure of rat and human adipose tissue to 0.25 to 5 Gy of γ-radiation increases the production of COX-2 and secretion of ATX and multiple inflammatory mediators [192]. This depends on DNA damage and the consequent activation of NF-κB and COX-2 signaling downstream of the activation of ATM serine/threonine kinase (ATM), ataxia telangiectasia and Rad3-related protein (ATR) and poly [ADP-ribose] polymerase 1 (PARP-1) [192] (Figure 3). Although radiation-induced activation of ATM increased the expressions of LPA_1_ and LPA_2_, the increases in ATX, COX-2 and inflammatory cytokines depends on activation of ATR [192]. These results show that these responses to radiation are activated by the accumulation of single-stranded DNA intermediates in double strand break resection during homologous recombination, rather than by double strand breaks themselves. The combined effects of radiation in causing inflammation in breast adipose tissue and thereby increasing the expression of ATX and signaling through LPA_1_ and LPA_2_ could increase the survival of residual breast cancer cells and decrease the effectiveness of the radiotherapy [192] (Figure 3). 

Furthermore, radiation-induced fibrosis is a significant contributor to long-term sequelae for cancer survivors. Blockage of LPA_1/3_ with the antagonist VPC12249 ameliorated radiation-induced fibrosis and radiation pneumonitis in murine models [196,197]. Taken together with the evidence that blocking LPA signaling can decrease the progression of pulmonary fibrosis, it is likely that inhibitors of LPA signaling could have a significant potential in decreasing the adverse side effects of radiation therapy as well as improving the killing of residual cancer cells. 

### 7.4. Inhibitors of LPA Signaling Entering into Clinical Trials 

The number of inhibitors against LPA signaling has now reached a critical mass, such that the first ATX and LPA receptor inhibitors have entered clinical trials [16] (Table 1). Galapagos NV have tested the first ATX inhibitor, GLPG1690, currently in Phase II trials for idiopathic pulmonary fibrosis (IPF) [198] (Table 1). In a blelomycin-induced pulmonary fibrosis murine model, an analog of GLPG1690 reduced extracellular matrix deposition in the lung by nearly 50% and reduced the concentrations 18:2-LPA in bronchoalveolar lavage fluid by nearly 70% [198]. Similarly, Bristol-Myers Squibb has put BMS-986020, a LPA_1_ antagonist, into Phase II trials for idiopathic pulmonary fibrosis [16,199]. Sanofi SAR100842, a LPA_1/3_ antagonist, is in Phase II trials for the treatment of systemic sclerosis and related fibrotic diseases [16,199]. 

In a non-fibrotic disease model, Lpath, Inc. developed an LPA-directed monoclonal antibody which lowered lesion volume in mouse models of traumatic brain injury by decreasing IL-6 levels and conducted a Phase I trial of the antibody as Lpathomab [16,200] (Table 1).

## 8. Potential Applications for Inhibitors of LPA Signaling

So far, there is no approved cancer therapy that targets the ATX-LPA-inflammatory axis, but this area opens the prospect of exciting new approaches to cancer treatment. It is unlikely that inhibiting LPA signaling alone will prove effective as a cancer mono-therapy. Also, from a pragmatic and ethical point of view, it would impossible to test a new mono-therapy for blocking LPA signaling where an accepted therapy already exists. We envisage that strategies for blocking LPA signaling will be used to improve the efficacies of existing chemotherapies and radiotherapy regimens. This is based on preclinical evidence that blocking the effects of LPA increases the efficacy of taxanes, cisplatin, tamoxifen and doxorubicin in treating cancers [201]. Equally, LPA protects cells from radiation-induced cell death [192,202]. Therefore, blocking inflammation and the radiation–induced expression of ATX should prevent a loss of efficacy of radiotherapy during subsequent fractions of radiation [192,202]. Eventual clinical trials in the cancer field are likely to study LPA signaling inhibitors as adjuvants to existing standard-of-care treatments, looking at standard parameters that measure response to primary treatment. If these inhibitors as adjuvants show benefit, future studies might look at dose reductions of the primary treatment agents (either chemotherapy or radiotherapy), which would potentially decrease cancer therapy side effects. 

One such group of major side effects is fatigue and the development of fibrosis secondary to radiotherapy. Fatigue is associated with the production of inflammatory cytokines during radiotherapy. Blocking LPA signaling should decrease this inflammatory response, which could also contribute to decreased scaring [203]. The positive effects of inhibiting ATX or LPA_1_ activation in treating pulmonary fibrosis provide encouraging evidence that these treatments could decrease radiation-induced fibrosis [196]. 

One of the major recent advances in cancer treatment has been the introduction of immunotherapy acting as checkpoint inhibitors. So far, there is no preclinical evidence that blocking LPA signaling will improve the efficacy of immunotherapies or combat its side effects. However, this is an intriguing possibility, since LPA and maladaptive inflammation contribute to immune evasion. The most frequent toxicities of immunotherapies include colitis, dermatitis, hepatitis, and pruritus [204,205], all conditions for which pre-clinical evidence exists that inhibition of LPA signaling might have therapeutic benefits.

## 9. Conclusions

In summary, we are at an exciting time where several therapeutics are in advanced clinical trials for blocking LPA signaling and inflammation. These agents are generally well tolerated and they could be tested as novel strategies for improving the effectiveness of existing cancer therapies. These approaches should be applicable to a wide variety of cancers since they target the tumor environment, which should be relatively independent of the specific mutation in the cancer cells. Overall, as mitigators of chronic inflammation, inhibitors of LPA signaling could become viable therapeutic modalities for preventing cancer initiation, maintaining the efficacy of chemotherapy and radiotherapy and prolonging remission. 

## Figures and Tables

**Table 1:** Summary of novel targets of the ATX-LPA signaling axis currently in clinical trials.

Compound	Mechanism of Action	Clinical Stage	Clinical Indication	Company	Clinicaltrials.gov ID
GLPG1690	ATX direct inhibition	Phase 2	IPF	Galapagos NV (Mechelen, Belgium)	NCT02738801
BMS-986020	LPA_1_ antagonist	Phase 2	IPF	Bristol-Myers Squibb (New York, NY, USA)	NCT01766817
SAR100842	LPA_1/3_ antagonist	Phase 2	Systemic Sclerosis	Sanofi (Paris, France)	NCT01651143
Lpathomab ^TM^	LPA monoclonal antibody	Phase 1	___	Lpath, Inc. (San Diego, CA, USA)	NCT02341508

IPF, idiopathic pulmonary fibrosis.
